# Psychiatric and non-psychiatric population vulnerabilities in time of a crisis: the unsuspected aggression factor

**DOI:** 10.1186/s12888-023-04843-4

**Published:** 2023-06-01

**Authors:** Sylvia Martin, Anna Oltra, Jonathan Del Monte

**Affiliations:** 1 Center for Research and Bioethics, Uppsala University, Husargatan 3, BMC, entrance A11, 75224 Uppsala, Sweden; 2 Psycho.Tcce, Clinical psychology Private practice, Montpellier, France; 3 Clinical psychology Private practice, Toulouse, France; 4 Psychosocial Laboratory, Aix-Marseille University, Aix Marseille, France; 5 Clinical Psychology Department, Nîmes University, Nîmes, France

**Keywords:** COVID-19, Quarantine, Anxiety, Depression, Impulsivity, Aggression

## Abstract

**Objectives:**

In March 2020, France faced a health crisis due to the COVID-19 outbreak that, like previous infectious disease crises, involved high psychological and emotional stress, a series of factors that influenced the ongoing mental health crisis.

**Methods:**

We recruited 384 respondents to complete an online questionnaire during the second month of isolation: 176 psychotherapy recipients (68 were currently attending psychiatric care) and 208 healthy controls. We measured demographic characteristics, impulsivity, aggression, hopelessness, suicidal risk, and the global level of anxiety and depression in order to estimate potential discrepancies in clinical measures across these populations.

**Results:**

Our results indicate that the group currently undergoing psychiatric care was prone to loneliness and social isolation. Regarding clinical and nonclinical population, there were differences in suicidal risk, depression, anxiety, and hopelessness but mainly in aggression. Regression analysis also demonstrated that aggression surprisingly influenced anxiety levels. Patients undergoing therapy compared with patients who were not displayed differences only in suicidal risk, anxiety, and hopelessness, with those undergoing therapy having higher scores. The outpatient group undergoing therapy had a significantly lower level of impulsivity. Moreover, the regression to predict anxiety and depression levels from correlated factors highlighted the potentially heightened role of aggression in predicting anxiety in the clinical group.

**Conclusion:**

New research into stress reactions should assess other clinical signals, such as aggression, and examine preventive mental health interventions in times of crisis.

**Supplementary Information:**

The online version contains supplementary material available at 10.1186/s12888-023-04843-4.

## Introduction

In March 2020, France faced the COVID-19 public health crisis. As we learned in previous pandemics (e.g., Ebola, SARS, and H1N1), the general population suffers from psychological stress and negative emotions during these crises [1, 2]. It is thus essential to monitor mental and behavioral factors that can influence health [3]. There is a lack of models predicting mass psychological reactions during disease outbreaks [4, 5].

During the COVID-19 crisis, most countries opted for lockdown measures that entailed separation from loved ones, loss of freedom, and negative outcomes such as inadequate access to supplies/resources, lack of information, and financial losses [6-12]. Researchers also stated that mental health prevention should support vulnerable individuals via telehealth [13].

### Anxiety and depression risks

COVID-19 studies have reported increased anxiety and depression levels in the general population across multiple countries from Asia to Europe [14-16]. Some research showed increased psychological discomfort [17] and even increased psychiatric symptoms among the general population, with, for example, 67% experiencing post-traumatic stress disorder (PTSD) and 19% suicidal ideation [18].

Fernandez et al. [19] looked at participants’ risk profiles, finding that, second to sociodemographic factors, preexisting psychiatric issues were associated with risk of psychological distress. In 2020, Iasevoli et al. [20] found that patients with serious mental illness were more likely to experience high pandemic-related stress and had a higher risk of experiencing more severe anxiety and depressive symptoms, suggesting that healthy controls might experience less psychological distress. Indeed, when measuring caregivers’ mean scores on the depression, perceived stress, and general anxiety disorder scales versus controls, Iasevoli et al. found a lower depression score and comparable stress and general anxiety disorder scores. The mean care givers self report score corresponded to mild burden.

Several researchers concluded that, second to demographic predictors, mental disorder history increases the risk effect of all mental health state indicators [21, 22]. To our knowledge, little research has assessed the anxiety and depression levels of mentally ill persons during COVID-19 in relation to aggression levels or impulsivity scores.

### What to fear?

An increased number of psychiatric care consultations was predicted in 2020 by psychiatrists [23], more so for patients with prior psychiatric diagnoses [24]. Many researchers recommended undisrupted care [25-27] as the vulnerable mentally ill were especially at risk of developing increased anxiety [28, 29]. A history of psychiatric illness was associated with experiencing anxiety and anger four to six months after release from isolation measures [30]. Jeong et al. showed that we could prevent mental health problems by providing support to individuals with vulnerable mental health, providing accurate information and appropriate supplies and accommodations during a health crisis. Very rapidly, experts recommended telehealth solutions to prevent some of the worst effects of mental health crises [31]. At first, the Chinese, Singaporean, and Australian governments noted the psychological side effects of COVID-19 that needed to be considered. *The Lancet* [28] alerted readers that the psychiatric population “might experience worsening symptoms”, whereas the general population “might develop new mental health problems, especially depression, anxiety, and post-traumatic stress (all factors associated with increased suicide risk)”.

Patients with identified psychiatric disorders (e.g., affective disorder, schizophrenia, and addictive disorders) reported heightened stress levels and half of them reported experiencing critical stress. A quarter of patients with affective disorders reported increased difficulties sleeping and increased irritability [29].

In a nonclinical population, decision-making impulsivity was thought likely to increase in several life domains, such as diet, high-risk behavior, and habits (e.g., smoking and legal and illegal substance use) [32]. People also experienced psychological conflict between the urge to feel safe and the desire for a pleasurable life, which resulted in maladaptive behaviors [33].

Some elements from the 2020’s literature filled us with impulsivity or aggressive outbursts fear that could be displayed by young adults or members of known impulsive populations [33-37]. Nivette et al. [33] feared that young adults displaying low trust and “antisocial potential” with previous low rule acceptance, low shame, and poor self-control (i.e., delinquents) would oppose or comply less with governmental measures. Populations with “dark personality” traits were thought likely to knowingly expose others to risks [34]. This vulnerability to impulsive behaviors also applied to the young adult population renowned for its proneness to gaming addiction. Verizon, an online game provider, noted a 70–75% increase in online gaming activity coinciding with initial stay-at-home directives [35, 38] and an increase in the number of active users to 20 million [39, 40].

Considering the adverse effects on depression and anxiety levels expected from the COVID-19 lockdowns and our initial observations as field clinicians, we hypothesized that participants with a history of psychiatric care would be more vulnerable than the general population and experience heightened psychological issues compared with the non-psychiatric population. Therefore, we chose to use questions about the context (i.e., sense of loneliness, impact of isolation on relational and emotional life, number of cohabitants during quarantine, and number of people the respondent was in contact with every week), clinical scales, and other scales found in the literature to confirm this hypothesis, assess the situation more precisely, and better understand the causes and consequences.

Our hypothesis is that the psychiatric population would experience more anxiety/depression, suicidal risk, aggression, and impulsivity than would the non-psychiatric population during the COVID-19 crisis.

## Methods

### Participants

We recruited 383 respondents to an online questionnaire using Google Forms during France’s second month of lockdown. The overall sample contained 110 students, four workers, 64 self-employed, 42 employees, 57 people with white-collar professions, 11 with non-specific status, 39 unemployed or on medical leave, 16 retirees, and 39 workman position. The gender ratio was 286 female, 95 male, and two nongendered participants. We recruited part of the sample from the general population, another part from private outpatient clinical care, and the last part from the Nîmes University health service. The participants completed clinical scales and supplied demographic data (20–30 min). The exclusion criteria for both groups were known neurological disease and developmental disability. All participants were proficient in the French language, had normal or corrected-to-normal vision, and were naive as to the purpose of the study. According to ethical provisions of the World Medical Association Code of Ethics (Helsinki Declaration) for experiments involving human subjects, participants gave online informed consent to participate in this experiment.

### Measures

The Beck Hopelessness Scale (BHS) from 1974 [41] was translated into French by Cottraux et al. in 1985 [42]. This scale is intended to evaluate pessimism and cognitive beliefs about the future, indirectly capturing suicidal intentions. Its items elicit binary true/false responses, with a total score ranging from 0 to 20. The Cronbach’s alpha of the instrument was 0.72.

The Suicide Behaviors Questionnaire – Revised (SBQ-R), formulated by Osman et al. in 2001 [43], assesses suicidal behaviors. SBQ-R is one of the few tools asking about anticipated future suicidal thoughts or actions as well as past and present ones; it includes items about lifetime suicidal ideation, plans to commit suicide, and actual attempts. Shakeri et al. [44] later reformulated it for a psychiatric population. A total score of 7 and higher in members of the general population or of 8 and higher in patients with psychiatric disorders indicates a significant risk of suicidal behavior. We used the validated French version of the instrument by Potard et al. [45]. The Cronbach’s alpha of the SBQ-R items was 0.80.

The Hospital Anxiety and Depression Scale (HADS) was created and validated by Zigmund and Snaith in 1983 and has been widely used in both general health care and psychiatric research [46]. The HADS identifies the presence of anxiety disorders and depression. It is divided into anxiety (HADS-A) and depression (HADS-D) subscales [47], having cut-off scores of 9 and 11, respectively; the Cronbach’s alpha was 0.67 for anxiety and 0.79 for depression.

The Aggression Questionnaire (AQ12), in its 2009 French translation by Genoud and Zimmermann [48], contains 12 items assessing the dimensions of aggression, but can be used to yield a single score. The questionnaires use a Likert scale ranging from 1 (“Not at all like me”) to 6 (“Completely like me”) and had a Cronbach’s alpha of 0.80.

Impulsive Behavior Scale – Short version (UPPS-S) was translated into French and validated by Billieux et al. in 2012 [49]. It consists of a self-report scale with 20 items assessing four factors of impulsivity: urgency (negative and positive), lack of premeditation, lack of perseverance, and sensation seeking. Positive urgency assesses impulsivity due to positive emotion, whereas negative urgency assesses impulsivity due to negative emotion. The respective Cronbach’s alphas indicated good consistency: negative urgency alpha = 0.78, positive urgency alpha = 0.70, lack of premeditation alpha = 0.79, lack of perseverance alpha = 0.84, and sensation seeking alpha = 0.83.

We formulated a questionnaire to collect demographic data regarding age, gender, and sociodemographic category. It also included the following questions: a) Have you ever been treated for psychological problems (by a psychologist or psychiatrist)? Response: *Yes/No*; b) Are you currently receiving professional psychological/psychiatric care (e.g., psychiatrist, psychologist, or psychological care structure such as daycare/hospital/ association)? Response: *Yes/No*; c) Do you feel more isolated/lonely at the moment (since confinement)? Response ranging from: *No, not at all* = *1* to *Yes, very often* = *5*; d) Since lockdown, with how many people have you had regular contact (at least twice a week, either face to face or via telephone or video-conference)?; e) How many people did you live with during confinement?; f) What has been the overall impact of confinement on your relationship life? Response ranging from: *Negative* = *1* to *Positive* = *5*; g) What has been the overall impact of the lockdown on your emotional life? Response ranging from: *Negative* = *1* to *Positive* = *5*.

### Group definitions

Our first defined group (Gp1) consisted of participants naive to psychiatric care (*n* = 201, 70% women, 29% men, 1% non-binary), the second group (Gp2) consisted of participants with past experience of such care (*n* = 114, 78% women, 21% men, 1% non-binary), and the third group (Gp3) consisted of current outpatient care recipients (*n* = 68, 79% women, 19% men, 1% non-binary). The nonclinical and clinical populations were recruited via social media and information about the research delivered to current patients of several clinical psychologists volunteering to recruit participants within their own networks. These three groups were created based on the questionnaire responses, allowing us to distinguish participants who had never had any experience of psychological/psychiatric care, participants who had receivedpsychiatric care at some point in life, but were not currently receiving it, and participants who reported having had experience of psychological/psychiatric care and were currently being supported by such treatment.

## Results

As the variables were normally distributed, we used Pearson parametric correlations to explore the relationships among the clinical data; we used multiple linear regression to estimate the shared covariance. For all analyses, the significance level was set to *p* < 0.05 (*), *p* < 0.005 (**), and *p* < 0.001 (***).

### Descriptive analysis

We used SPSS 2.0 software for descriptive analysis, correlation analysis, and regression observation, whereas additional analyses were performed using R. For observational purposes, we ran several sample analyses. Demographic measure means are presented in Table 1 and clinical measure means are presented in Table 2.

**Table 1:** Means and standard deviations for our sample

	Group (short description)	Mean	SD
age	Gp1(Naive)	32.43	14.03
	Gp2(Past)	33.57	12.19
	Gp3(Current)	36.25	11.94
Sense of Loneliness	Gp1(Naive)	2.69	1.26
	Gp2(Past)	2.76	1.25
	Gp3(Current)	3.18	1.19
N° Contact	Gp1(Naive)	8.21	7.47
	Gp2(Past)	9.32	11.51
	Gp3(Current)	7.21	6.25
Number of cohabitants	Gp1(Naive)	2.12	1.68
	Gp2(Past)	1.93	1.54
	Gp3(Current)	1.46	1.54
Relational	Gp1(Naive)	2.86	0.92
	Gp2(Past)	2.90	0.93
	Gp3(Current)	2.77	0.91
Emotional	Gp1(Naive)	2.72	1.00
	Gp2(Past)	2.86	1.11
	Gp3(Current)	2.64	1.18

*Note*: *N° contact* number of persons in contact with, *Relational* relational life perceived impact, *Emotional* emotional life perceived impact, *Gp1(Naive)* participants naive to psychological care, *Gp2 (Past)* participants with past experiences of psychological care, *Gp3 (Current)* Out care patients

**Table 2:** Clinical measure’s means

	Group	Mean	SD
SBQ-R	Gp1	5.22	2.512
	Gp2	6.44	3.204
	Gp3	8.59	4.268
HADS-A	Gp1	8.23	4.160
	Gp2	9.50	3.872
	Gp3	11.13	4.63
HADS-D	Gp1	5.00	3.57
	Gp2	5.65	3.72
	Gp3	6.87	4.31
Negative Urgency	Gp1	13.20	5.77
	Gp2	14.64	6.09
	Gp3	12.81	5.90
Positive Urgency	Gp1	11.76	3.90
	Gp2	11.90	3.99
	Gp3	11.26	3.68
Lack of Premeditation	Gp1	8.67	4.13
	Gp2	9.24	4.10
	Gp3	8.54	3.80
Lack of Perseverances	Gp1	7.23	2.65
	Gp2	7.64	2.67
	Gp3	8.57	3.12
Sensation Seeking	Gp1	9.84	2.89
	Gp2	9.78	2.95
	Gp3	9.66	3.42
BHS	Gp1	5.59	4.18
	Gp2	6.56	4.26
	Gp3	7.88	5.14
AQ12	Gp1	27.84	9.37
	Gp2	31.36	10.31
	Gp3	32.23	10.95

*Note*: *BHS* Hopelessness, *AQ12* Aggression, *Gp1* participants naive to psychological care, *Gp2* participants with past experiences of psychological care, *Gp3* Out care patients

### Comparative analysis

**Fig. 1:**
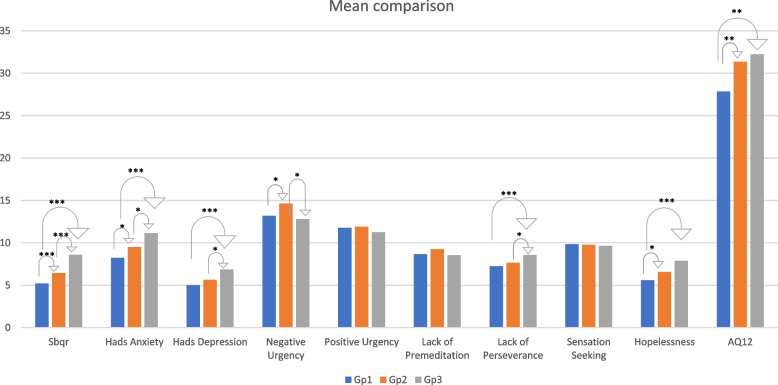
Means comparisons

We ran *t*-test analyses comparing Gp1 and Gp2, finding significant differences (*t* [313]) in SBQ-R (*p* = ***), HADS-A (*p* = *), negative urgency (*p* = *), BHS (*p* = *), and AQ12 (*p* = **). For the comparison of Gp1 with Gp3, we found significant differences (*t* [267]) in age (*p* = *), sense of loneliness (*p* = *), number of persons one is isolated with (*p* = *), lack of perseverance (*p* = ***), BHS (*p* = ***), HADS-D (*p* = ***), HADS-A (*p* = ***), SBQ-R (*p* = ***), and AQ12 (*p* = **). For the Gp2 and Gp3 comparison, there were significant *t*-test results for SBQ-R (*p* = ***), sense of loneliness (*p* = *), HADS-D (*p* = *), HADS-A (*p* = *), negative urgency (*p* = *), and lack of perseverance (*p* = *). All results are summarized in Table 3 and Fig. 1.

**Table 3:** T-test comparisons

Gp1&2					Gp2&3				Gp3&1			
	**t**	**df**	***p***	**Cohen’s** **d**	**t**	**df**	***p***	**Cohen’s d**	**t**	**df**	***p***	**Cohen’s** **d**
age	-0.724	313	0.470	-0.085	-1.446	180	0.150	-0.222	-2.010	267	**0.045**	-0.282
Sense of loneliness	-0.408	268	0.683	-0.052	-2.101	154	**0.037**	-0.346	-2.632	232	**0.009**	-0.394
Ncontact	-0.963	269	0.336	-0.122	1.312	156	0.191	0.214	0.931	233	0.353	0.139
Ncohabitants	0.934	269	0.351	0.118	1.859	156	0.065	0.304	2.705	233	**0.007**	0.403
Relational life	-0.335	268	0.738	-0.043	0.849	154	0.397	0.140	0.651	232	0.516	0.098
Emotional life	-1.021	252	0.308	-0.134	1.138	144	0.257	0.194	0.498	216	0.619	0.078
SBQ-R	-3.739	313	** < .001**	-0.438	-3.858	180	** < .001**	-0.591	-7.876	267	** < .001**	-1.105
HADS-A	-2.671	313	**0.008**	-0.313	-2.555	180	**0.011**	-0.392	-4.834	267	** < .001**	-0.678
HADS-D	-1.515	313	0.131	-0.178	-2.013	180	**0.046**	-0.308	-3.521	267	** < .001**	-0.494
Negative Urgency	-2.079	313	**0.038**	-0.244	1.984	180	**0.049**	0.304	0.485	267	0.628	0.068
Positive Urgency	-0.309	313	0.758	-0.036	1.076	180	0.283	0.165	0.921	267	0.358	0.129
Lack of Premeditation	-1.171	313	0.243	-0.137	1.133	180	0.259	0.174	0.225	267	0.822	0.032
Lack of Perseverance	-1.303	313	0.194	-0.153	-2.141	180	**0.034**	-0.328	-3.437	267	** < .001**	-0.482
Sensation Seeking	0.162	313	0.872	0.019	0.248	180	0.805	0.038	0.410	267	0.682	0.057
BHS	-1.975	313	**0.049**	-0.232	-1.871	180	0.063	-0.287	-3.685	267	** < .001**	-0.517
AQ12	-3.086	313	**0.002**	-0.362	-0.542	180	0.589	-0.083	-3.198	267	**0.002**	-0.449

*Note.* Student’s t-test

^a^Levene’s test is significant (*p* < .05), suggesting a violation of the equal variance assumption

For further insight, we compared the present scores with scores from the literature and clinical thresholds, as presented in Table 4. These results led us to further analyze the correlation within these groups.

**Table 4:** Comparison with the literature from previous research (before COVID)

Scale	Results from the present research	Differences	Literature scores	N	Cohen’s d
SBQ-R	Clinical (Gp3)	** < **	**Clinical**	69	**1.01**
	**Non clinical (Gp1)**	>	Non Clinical	120	.09
HADS-A	**Clinical (Gp3) + **	>	Clinical +	491	.03
	**Non clinical (Gp1) + **	>	Non Clinical	1792	.05
HADS-D	Clinical (Gp3)	** < **	**Clinical + **	491	**.46**
	**Non clinical (Gp1)**	** > **	Non Clinical	1792	**.40**
Negative Urgency	Clinical (Gp3) +	<	**Clinical + **	81	.1
	**Non clinical (Gp1) + **	** > **	Non Clinical	650	**.74**
Positive Urgency	Clinical (Gp3) +	** < **	**Clinical + **	268	**.4**
	**Non clinical (Gp1) + **	** > **	Non Clinical	650	**.27**
Lack of Premeditation	Clinical (Gp3) +	** < **	**Clinical + **	268	**.34**
	**Non clinical (Gp1) + **	>	Non Clinical	650	.21
Lack of Perseverance	Clinical (Gp3)	<	**Clinical + **	268	.16
	Non clinical (Gp1)	<	**Non Clinical**	650	.09
Sensation Seeking	Clinical (Gp3)	** < **	**Clinical + **	81	**.30**
	**Non clinical (Gp1)**	** > **	Non Clinical	650	.25
Hopelesness	Clinical (Gp3) +	** < **	**Clinical + **	340	.20
	**Non clinical (Gp1) + **	** > **	Non Clinical	100	.02
AQ12	Clinical (Gp1) +	** < **	**Clinical + **	107	**.26**
	**Non Clinical (Gp3)**	** > **	Non Clinical	101	**.94**

*Note*: < **or >  = **cohen’s d higher than .25; < **or >  = **cohen’s d lower then. 25, Non clinical sample: *n* = 201, Clinical sample: *n* = 68, AQ12 scores extracted from Vitoratou et al. [50], Hopelessness scale scores extracted from Szabó et al. [51], UPPS scores extracted from Martin et al. [52]; Hads scores extracted from Crawford et al. [53] and Spinhoven et al. [54]; Sbqr scores extracted from Osman et al. [42] and Au et al. [55]; + : above pathological sthresolds

### Correlations

#### In the nonclinical sample (group 1)

HADS-A was correlated to sense of loneliness (*r* = 0.289**), perceived impact on relational life (*r* = –0.250**), perceived impact on emotional life (*r* = –0.381**), SBQ-R (*r* = 0.224**), BHS (*r* = 0.404**), AQ12 (*r* = 0.486**), negative urgency (*r* = 0.255**), positive urgency (*r* = 0.306**), HADS-D (*r* = 0.520***), and lack of premeditation (*r* = 0.283**).

For HADS-D, correlations were found with sense of loneliness (*r* = 0.261**), perceived impact on relational life (*r* = –0.307**), perceived impact on emotional life (*r* = –0.398**), SBQ-R (*r* = 0.353**), BHS (*r* = 0.516**), AQ12 (*r* = 0.469**), negative urgency (*r* = 0.170*), lack of perseverance (*r* = 0.242**), and lack of premeditation (*r* = 0.268*).

Aside from the above-mentioned correlations, AQ12 was correlated with age (*r* = 0.211**), perceived impact on emotional life (*r* = –0.258**), negative urgency (*r* = 0.272***), positive urgency (*r* = 0.224**), lack of premeditation (*r* = 0.283***), lack of perseverance (*r* = 0.183*), and H (*r* = 0.444***).

#### In patients with experience of past therapy (group 2)

HADS-A was correlated with age (*r* = –0.233*), number of persons isolated with (*r* = 0.258*), SBQ-R (*r* = 0.259**), H (*r* = 0.341**), AQ12 (*r* = 0.344**), HADS-A (*r* = 0.355***), and positive urgency (*r* = 0.202*).

HADS-D was correlated with sense of loneliness (*r* = 0.376**), SBQ-R (*r* = 0.297**), BHS (*r* = 0.454**), AQ12 (*r* = 0.236*), lack of premeditation (*r* = 0.257**), and lack of perseverance (*r* = 0.245**).

For AQ12, adding to its correlation with both HADS dimensions, we noted significant correlations with age (*r* = –0.246*), number of persons in contact with (*r* = –0.202*), SBQ-R (*r* = 0.232*), sensation seeking (*r* = 0.201*), and BHS (*r* = 0.264**).

#### In patients with current therapy (group 3)

HADS-A was correlated with sense of loneliness (*r* = 0.417**), impact on relational life (*r* = –0.315*), impact on emotional state (*r* = –0.494**), SBQ-R (*r* = 0.432**), BHS (*r* = 0.440**), HADS-D (*r* = 0.676***), and AQ12 (*r* = 0.516**).

For HADS-D, correlations were found with sense of loneliness (*r* = 0.337**), impact on relational life (*r* = –0.442**), impact on emotional state (*r* = –0.494**), SBQ-R (*r* = 0.423**), BHS (*r* = 0.649**), AQ12 (*r* = 0.300*), and lack of premeditation (*r* = 0.268*).

For AQ12, as well as the above-mentioned correlations with HADS-A and HADS-D, there were significant correlations with age (*r* = –0.317*), perceived impact on emotional life (*r* = 0.412**), SBQ-R (*r* = 0.250*), negative urgency (*r* = 0.266*), and BHS (*r* = 0.303*).

The correlation tables for all three groups are available as Supplementary Material 1 (Tables A, B, and C).

### Regression analysis

For Gp1, representing participants who had never received psychological care, we ran a regression analysis to see which factors predicted HADS-A. Four dimensions predicted anxiety levels, i.e., BHS (*p* = 0.035, *β* = 0.159), AQ12 (*p* = 0.008, *β* = 0.310), HADS-D (*p* = 0.035, *β* = 0.360), and positive urgency (*p* = 0.012, *β* = 0.178), with *R*^2^ = 0.500 and *F* = 15.209.

For HADS-D, there were four predictive dimensions, i.e., relational life (*p* = 0.037, *β* = –0.151), BHS(*p* = 0.015, *β* = 0.187), HADS-A (*p* = 0.000, *β* = 0.340), and AQ12 (*p* = 0.030, *β* = 0.165), with *R*^2^ = 0.487 and *F* = 16.148.

For AQ12, there were four predictive factors, i.e., HADS-D (*p* = 0.015, *β* = 0.173), SBQ-R (*p* = 0.000, *β* = 0.245), HADS-A (*p* = 0.001, *β* = 0.249), and age (*p* = 0.007, *β* = –0.192), with *R*^2^ = 0.45 and *F* = 12.790.

For patients with past therapy (Gp2): HADS-A was predicted by AQ12 (*p* = 0.019, *β* = 0.221), BHS (*p* = 0.041, *β* = 0.213), and number of cohabitants (*p* = 0.012, *β* = 0.230), with *R*^2^ = 0.313 and *F* = 8.289; HADS-D was predicted by BHS (*p* = 0.013, *β* = 0.277) and sense of loneliness (*p* = 0.004, *β* = –0.284), with *R*^2^ = 0.337 and *F* = 7.532; and AQ12 was predicted by only HADS-A (*p* = 0.017, *β* = 0.256) and sensation seeking (*p* = 0.005, *β* = 0.276), with *R*^2^ = 0.273 and *F* = 4.773.

For Gp3, representing patients currently receiving therapy, HADS-A was predicted by HADS-D (*p* = 0.002, *β* = 0.437) and AQ12 (*p* = 0.001, *β* = 0.363), with *R*^2^ = 0.637 and *F* = 11.776, whereas HADS-D was predicted by HADS-A (*p* = 0.002, *β* = 0.430) and BHS (*p* = 0.003, *β* = 0.370), with *R*^2^ = 0.658 and *F* = 0.9.628.

**Fig. 2:**
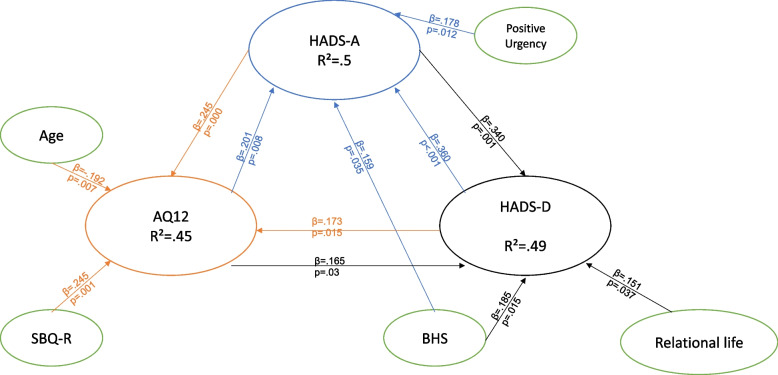
Regression array in Gp1

**Fig. 3:**
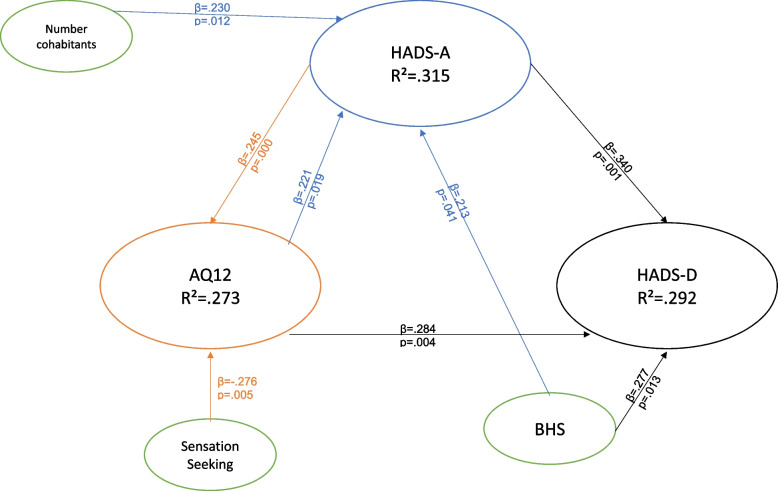
Regressions array for Gp2

**Fig. 4:**
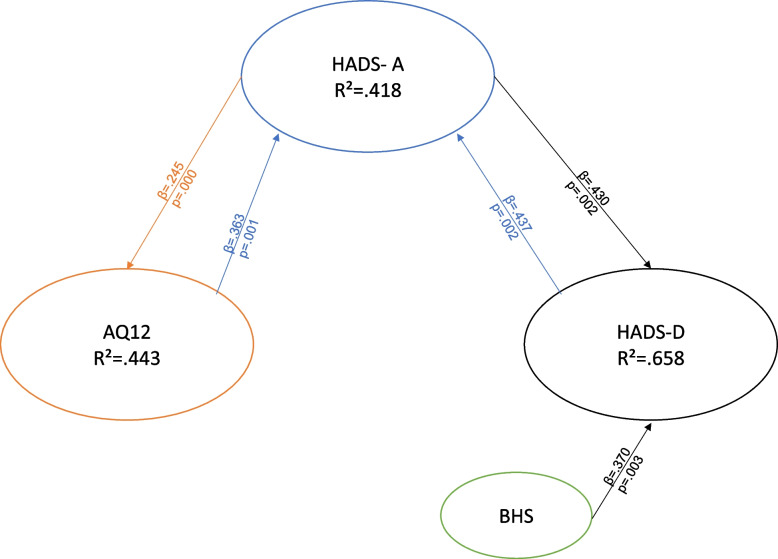
Regression array for Gp3

For a graphical overview of the three regression arrays depending on the group, see Figs. 2, 3 and 4. The regression tables are available as Supplementary Material 2 (Tables D, E, and F).

## Discussion

Our results are globally congruent with the literature concerning the psychological impact of the COVID-19 pandemic. In the first pandemic wave, increased stress and anxiety levels were demonstrated in the general population, and stress-sensitive populations were particularly at risk [56]. Comparing our results with norms and potential scores from the literature referring to comparable populations, we found that the nonclinical participants usually experienced scores above the pathological thresholds, calling into question their psychological well-being during quarantine.

Our specific HADS score results indicated elevated anxiety and depression rates, mainly for anxiety levels among outpatient care recipients during the COVID-19 crisis. All correlated dimensions in this group appeared elevated compared with those of other groups, i.e., for the hopelessness, suicidal thoughts, aggression, and some impulsivity dimensions. Regarding demographic outcomes, respondents currently receiving care also had fewer cohabitants than did respondents not receiving therapy and felt significantly lonelier during quarantine.

For HADS-A, the only common predictive factor in all three groups’ regressions was aggression. Some previous studies have noted the role of aggression in affecting stress and anxiety levels [57-60]. Research has also demonstrated that depression and suicidality are related to aggression [61-63]. Other research has even related to aggression [64, 65] or focused on predicting personality disorder through aggression [66-68]. All these findings tend to question whether aggression has impacts on global health in the general population [69-71], which could open the way for further studies. So far, few researchers have taken an interest in measuring aggression levels to estimate other complex dimensions; for example, Vora et al. [72] and Anurudran et al. [73] predicted substance abuse and behavioral disorders (i.e., domestic violence and child abuse) during COVID-19 quarantines. As our results lack causal significance, further research is needed to determine the direction of the discovered interrelated prediction models.

For HADS-D, the only common factor throughout our different groups was hopelessness and HADS-A. The importance of hopelessness for psychological distress was already noted in the literature. Some research found that anxiety levels were predictors of hopelessness [74, 75]. However, researchers found only a moderate increase in hopelessness levels compared with pre-pandemic levels [76]. This observation confirmed the strong relationship of this dimension with anxiety and depression levels [77]. In young adults, we expected to observe stress and anger levels related to hopelessness, as did Shanahan et al. [78].

Globally, we noticed, as did Galea et al. [79], the insufficient research on the mental health consequences of epidemics versus other kinds of disasters. Catastrophic events sometimes increase depression and/or anxiety levels and increase the risk of developing PTSD. Greater research into the adverse mental health effects of pandemics would appear necessary in order to formulate adequate psychological care policies for times of crisis.

In France, Chaix et al. [80] found that patients with depression had a 53% increased risk of developing psychological distress during the first lockdown. Essadek and Rabeyron [81] evaluated the impact of the pandemic on French students, showing that they suffered from high levels of anxiety, depression, and distress and that students might need special attention in terms of psychological support during the pandemic.

Although we should pay attention to the population at risk during pandemics, other studies suggest that lockdowns could affect the mental health of the general French population as well [82]. This impact has been deduced from research on the increase in consumption of antidepressants and antipsychotics, by 21.6% and 21.5%, respectively, during the lockdown [83]. On the other hand, however, Pham Scottez et al. [84] showed a significant decrease in psychiatric emergency traffic in a French consultation ward in 2020 compared with pre-pandemic years. This decrease could be explained by patients’ decisions to postpone consultations because of fear of the virus. This suggests another reason to encourage the care of vulnerable populations during pandemics and to adopt effective strategies to reduce the adverse psychological effects of lockdowns.

### Limitations

We did not control for any precise psychopathological diagnoses, as this was an online questionnaire and we decided to protect medical privacy. The second limitation comes from collecting our data during the second month of lockdown. Third, we lack baseline data and therefore insight into the evolution of the respondents’ scores from the pre-pandemic period, and comparison with previous scores from the literature cannot be considered sufficient to draw any conclusions. Finally, as the gender ratio in all groups was above 70% women, the results would be difficult to generalize to more evenly mixed samples..

## Conclusion

Our research examines the underlying vulnerabilities that emerge in a time of crisis from unsuspected dimensions across different more or less psychologically fragile populations. During lockdown, we found higher levels of anxiety, depression, hopelessness, aggression, and some impulsivity in a group currently in therapy (Grp 3) than in the control group from the general population (Grp 1). The vulnerable population (group 2 and 3) was prone to loneliness and was socially isolated during lockdown periods. Surprisingly, the group naïve to psychotherapy (Grp 1), supposedly less vulnerable to psychological distress, was also affected by diverse stress factors, attaining high, clinically significant scores on six out of ten measured clinical dimensions.

In both clinical and nonclinical samples, the only common factor predicting HADS-A was aggression; for HADS-D, both samples shared hopelessness as a predictive factor.

These results call for a better understanding of the factors leading to adverse psychological effects during pandemic and lockdown periods. This will permit us to assess the at-risk population and suggest adequate responses, preventing long-term adverse consequences such as PTSD symptoms, isolation, suicidal risk, and increased domestic violence. Further research is needed to better understand the dynamics in play between aggression and anxiety levels across different groups, to potentially develop preventive actions to relieve aggression and anxiety.

## Supplementary Information

**Additional file 1:** **Table A. **Correlation table for Gp1. **Table B. **Correlation table for Gp2.** Table C. **Correlation table for Gp3.

**Additional file 2:** **Table D. **Regression intra Gp1. **Table E. **Regression intra Gp2. **Table F.** Regression intra Gp 3.

**Additional file 3:** Complementary analysis.

## Data Availability

Data are available from the corresponding author on reasonable request.
