# Supplementary material for: Psychiatric and non-psychiatric population vulnerabilities in time of a crisis: the unsuspected aggression factor
Source: BMC Psychiatry. 2023 Jun 1;23:386. doi: 10.1186/s12888-023-04843-4 (PMC10234249; doi:10.1186/s12888-023-04843-4)
Supplement: Supplementary file 1 — Additional file 1: Table A. Correlation table for Gp1. Table B. Correlation table for Gp2. Table C. Correlation table for Gp3. [file 12888_2023_4843_MOESM1_ESM.docx]

**Table A : Correlation table for Gp1:**

|  | | age | isolation | Number  contact | NumberPers  .isolated with | relation | emotion | Sbqr | hadsAnx | hadsDep | NegUrg | PosUrg | LackPem | LackPers | Sensation seeking | H | AQ12 |
| --- | --- | --- | --- | --- | --- | --- | --- | --- | --- | --- | --- | --- | --- | --- | --- | --- | --- |
| hadsAnx | Pearson | -,045 | ,289^***^ | ,016 | ,060 | -,250^**^ | -,381^***^ | ,224^**^ | 1 | ,520^***^ | ,255^**^ | ,306^***^ | ,283^***^ | ,069 | -,066 | ,404^***^ | ,486^***^ |
|  | Sig. | ,523 | ,000 | ,831 | ,434 | ,001 | ,000 | ,001 |  | ,000 | ,000 | ,000 | ,000 | ,331 | ,355 | ,000 | ,000 |
| hadsDep | Pearson | ,008 | ,261^**^ | -,039 | -,053 | -,307^***^ | -,398^***^ | ,353^***^ | ,520^***^ | 1 | ,170^**^ | ,120 | ,115 | ,242^**^ | ,020 | ,516^***^ | ,469^***^ |
|  | Sig. | ,910 | ,001 | ,613 | ,488 | ,000 | ,000 | ,000 | ,000 |  | ,016 | ,089 | ,104 | ,001 | ,778 | ,000 | ,000 |
| AQ12 | Pearson | -,211^**^ | ,136 | -,055 | ,091 | -,076 | -,258^**^ | ,400^***^ | ,486^***^ | ,469^***^ | ,272^***^ | ,224^**^ | ,283^***^ | ,183^*^ | ,030 | ,444^***^ | 1 |
|  | Sig. | ,003 | ,073 | ,471 | ,235 | ,321 | ,001 | ,000 | ,000 | ,000 | ,000 | ,001 | ,000 | ,009 | ,675 | ,000 |  |

Note: isolation: sense of loneliness, Numbercontact=number of people being in contact with, relation =impact on relational life, emotion=impact on emotional life, NegUrg=Negative Urgency, PosUrg=Positive Urgency, LackPrem=Lack of premeditation, LackPers=Lack of perseverance, H=Hopelessness, AQ12= Aggression score, p<.05=*, p<.005=**,p<.001=***.

**Table B: Correlation table for Gp2:**

| **:** | | age | isolation | Number  contact | NumberPers  .isolated with | relation | emotion | Sbqr | hadsAnx | hadsDep | NegUrg | PosUrg | LackPem | LackPers | Sensation seeking | H | AQ12 |
| --- | --- | --- | --- | --- | --- | --- | --- | --- | --- | --- | --- | --- | --- | --- | --- | --- | --- |
| hadsAnx | Pearson | -,233^*^ | ,187 | -,016 | ,258^*^ | -,104 | -,154 | ,259^**^ | 1 | ,355^***^ | ,154 | ,202^*^ | ,141 | ,038 | -,116 | ,341^***^ | ,344^***^ |
|  | Sig. | ,013 | ,068 | ,879 | ,011 | ,314 | ,146 | ,005 |  | ,000 | ,103 | ,031 | ,134 | ,688 | ,220 | ,000 | ,000 |
| hadsDep | Pearson | -,165 | ,376^***^ | -,098 | ,074 | -,189 | -,188 | ,297^**^ | ,355^***^ | 1 | ,050 | ,161 | ,257^*^ | ,245^*^ | -,093 | ,454^***^ | ,236^*^ |
|  | Sig. | ,079 | ,000 | ,342 | ,470 | ,065 | ,075 | ,001 | ,000 |  | ,599 | ,087 | ,006 | ,009 | ,327 | ,000 | ,011 |
| AQ12 | Pearson | -,246^*^ | -,115 | -,202^*^ | ,094 | -,050 | -,108 | ,232^*^ | ,344^***^ | ,236^*^ | ,141 | ,117 | ,098 | ,142 | ,201^*^ | ,264^**^ | 1 |
|  | Sig. | ,008 | ,266 | ,048 | ,361 | ,626 | ,306 | ,013 | ,000 | ,011 | ,136 | ,213 | ,299 | ,131 | ,032 | ,005 |  |

Note: isolation: sense of loneliness, Numbercontact=number of people being in contact with, relation =impact on relational life, emotion=impact on emotional life, NegUrg=Negative Urgency, PosUrg=Positive Urgency, LackPrem=Lack of premeditation, LackPers=Lack of perseverance, H=Hopelessness, AQ12= Aggression score, p<.05=*, p<.005=**,p<.001=***.

**Table C: Correlation table for Gp3**

|  | | age | isolation | Number  contact | NumberPers  .isolated with | relation | emotion | Sbqr | hadsAnx | hadsDep | NegUrg | PosUrg | LackPem | LackPers | Sensation seeking | H | AQ12 |
| --- | --- | --- | --- | --- | --- | --- | --- | --- | --- | --- | --- | --- | --- | --- | --- | --- | --- |
| hadsAnx | Pearson | -,148 | ,417^***^ | -,078 | ,243 | -,315^*^ | -,494^***^ | ,432^***^ | 1 | ,676^***^ | ,099 | ,150 | ,223 | ,154 | ,030 | ,440^***^ | ,516^***^ |
|  | Sig. | ,229 | ,001 | ,553 | ,059 | ,014 | ,000 | ,000 |  | ,000 | ,423 | ,223 | ,067 | ,210 | ,807 | ,000 | ,000 |
| hadsDep | Pearson | ,002 | ,337^**^ | -,226 | ,172 | -,442^***^ | -,494^***^ | ,423^***^ | ,676^***^ | 1 | ,141 | ,050 | ,268^*^ | ,278^*^ | -,155 | ,649^***^ | ,300^*^ |
|  | Sig. | ,988 | ,009 | ,079 | ,185 | ,000 | ,000 | ,000 | ,000 |  | ,252 | ,684 | ,027 | ,022 | ,207 | ,000 | ,013 |
| AQ12 | Pearson | -,317^*^ | ,156 | ,033 | ,186 | -,069 | -,412^**^ | ,250^*^ | ,516^***^ | ,300^*^ | ,266^*^ | ,151 | ,070 | ,173 | ,009 | ,303^*^ | 1 |
|  | Sig. | ,009 | ,233 | ,803 | ,151 | ,601 | ,002 | ,040 | ,000 | ,013 | ,028 | ,218 | ,572 | ,159 | ,945 | ,012 |  |

Note: isolation: sense of loneliness, Numbercontact=number of people being in contact with, relation =impact on relational life, emotion=impact on emotional life, NegUrg=Negative Urgency, PosUrg=Positive Urgency, LackPrem=Lack of premeditation, LackPers=Lack of perseverance, H=Hopelessness, AQ12= Aggression score, p<.05=*, p<.005=**,p<.001=***.
