# Supplementary material for: Psychiatric and non-psychiatric population vulnerabilities in time of a crisis: the unsuspected aggression factor
Source: BMC Psychiatry. 2023 Jun 1;23:386. doi: 10.1186/s12888-023-04843-4 (PMC10234249; doi:10.1186/s12888-023-04843-4)
Supplement: Supplementary file 2 — Additional file 2: Table D. Regression intra Gp1. Table E. Regression intra Gp2. Table F. Regression intra Gp 3. [file 12888_2023_4843_MOESM2_ESM.docx]

| \|  \| **AQ12** \| \| \| \| --- \| --- \| --- \| --- \| \| *Predictors* \| *Estimates* \| *CI* \| *p* \| \| (Intercept) \| 15.00 \| 7.20 – 22.79 \| **<0.001** \| \| hadsDep \| 0.51 \| 0.10 – 0.92 \| **0.015** \| \| age \| -0.12 \| -0.21 – -0.03 \| **0.007** \| \| emotion \| -0.08 \| -1.36 – 1.19 \| 0.899 \| \| PosUrg \| -0.21 \| -0.55 – 0.14 \| 0.239 \| \| NegUrg \| 0.13 \| -0.11 – 0.37 \| 0.274 \| \| LackPrem \| 0.29 \| -0.03 – 0.61 \| 0.075 \| \| LackPers \| 0.18 \| -0.27 – 0.63 \| 0.428 \| \| Sbqr \| 1.07 \| 0.58 – 1.56 \| **<0.001** \| \| hadsAnx \| 0.63 \| 0.25 – 1.00 \| **0.001** \| \| R^2^ / R^2^ adjusted \| 0.450 / 0.418 \| \| \| | \|  \| **hadsDep** \| \| \| \| --- \| --- \| --- \| --- \| \| *Predictors* \| *Estimates* \| *CI* \| *p* \| \| (Intercept) \| 1.79 \| -1.59 – 5.17 \| 0.298 \| \| AQ12 \| 0.06 \| 0.01 – 0.12 \| **0.030** \| \| emotion \| -0.39 \| -0.96 – 0.18 \| 0.175 \| \| relation \| -0.59 \| -1.15 – -0.04 \| **0.037** \| \| NegUrg \| -0.02 \| -0.10 – 0.06 \| 0.638 \| \| LackPers \| 0.14 \| -0.03 – 0.31 \| 0.104 \| \| Sbqr \| 0.06 \| -0.14 – 0.27 \| 0.530 \| \| hadsAnx \| 0.31 \| 0.17 – 0.44 \| **<0.001** \| \| H \| 0.17 \| 0.03 – 0.30 \| **0.015** \| \| isolation \| -0.10 \| -0.53 – 0.33 \| 0.643 \| \| R^2^ / R^2^ adjusted \| 0.487 / 0.457 \| \| \| \|  \|  \| \| \| | \|  \| **hadsAnx** \| \| \| \| --- \| --- \| --- \| --- \| \| *Predictors* \| *Estimates* \| *CI* \| *p* \| \| (Intercept) \| 2.35 \| -1.34 – 6.04 \| 0.210 \| \| AQ12 \| 0.09 \| 0.02 – 0.15 \| **0.008** \| \| Sbqr \| -0.18 \| -0.40 – 0.05 \| 0.122 \| \| hadsDep \| 0.40 \| 0.24 – 0.57 \| **<0.001** \| \| NegUrg \| -0.02 \| -0.12 – 0.08 \| 0.671 \| \| PosUrg \| 0.18 \| 0.04 – 0.32 \| **0.012** \| \| emotion \| -0.38 \| -1.01 – 0.25 \| 0.234 \| \| relation \| -0.14 \| -0.76 – 0.48 \| 0.653 \| \| isolation \| 0.18 \| -0.29 – 0.65 \| 0.455 \| \| LackPrem \| 0.08 \| -0.05 – 0.21 \| 0.234 \| \| H \| 0.16 \| 0.01 – 0.31 \| **0.035** \| \| R^2^ / R^2^ adjusted \| 0.500 / 0.467 \| \| \| |
| --- | --- | --- | --- | --- | --- | --- | --- | --- | --- | --- | --- | --- | --- | --- | --- | --- | --- | --- | --- | --- | --- | --- | --- | --- | --- | --- | --- | --- | --- | --- | --- | --- | --- | --- | --- | --- | --- | --- | --- | --- | --- | --- | --- | --- | --- | --- | --- | --- | --- | --- | --- | --- | --- | --- | --- | --- | --- | --- | --- | --- | --- | --- | --- | --- | --- | --- | --- | --- | --- | --- | --- | --- | --- | --- | --- | --- | --- | --- | --- | --- | --- | --- | --- | --- | --- | --- | --- | --- | --- | --- | --- | --- | --- | --- | --- | --- | --- | --- | --- | --- | --- | --- | --- | --- | --- | --- | --- | --- | --- | --- | --- | --- | --- | --- | --- | --- | --- | --- | --- | --- | --- | --- | --- | --- | --- | --- | --- | --- | --- | --- | --- | --- | --- | --- | --- | --- | --- | --- | --- | --- | --- | --- | --- | --- | --- | --- | --- | --- | --- | --- | --- | --- | --- | --- | --- | --- | --- | --- | --- | --- | --- | --- | --- | --- | --- | --- |

**Table D: Regression intra Gp1:**

Note: isolation: sense of loneliness, Numbercontact=number of people being in contact with, relation =impact on relational life, emotion=impact on emotional life, NegUrg=Negative Urgency, PosUrg=Positive Urgency, LackPrem=Lack of premeditation, LackPers=Lack of perseverance, H=Hopelessness, AQ12= Aggression score, p<.05=*, p<.005=**,p<.001=***.

| \|  \| **AQ12** \| \| \| \| --- \| --- \| --- \| --- \| \| *Predictors* \| *Estimates* \| *CI* \| *p* \| \| (Intercept) \| 13.87 \| 1.15 – 26.60 \| **0.033** \| \| hadsDep \| 0.03 \| -0.56 – 0.63 \| 0.913 \| \| age \| -0.11 \| -0.28 – 0.07 \| 0.218 \| \| Sbqr \| 0.10 \| -0.57 – 0.77 \| 0.768 \| \| hadsAnx \| 0.70 \| 0.13 – 1.27 \| **0.017** \| \| NumberIsolated \| 0.27 \| -1.06 – 1.59 \| 0.692 \| \| H \| 0.50 \| -0.07 – 1.08 \| 0.082 \| \| Sensationseeking \| 0.97 \| 0.31 – 1.64 \| **0.005** \| \| R^2^ / R^2^ adjusted \| 0.273 / 0.216 \| \| \| | \|  \| **hadsDep** \| \| \| \| --- \| --- \| --- \| --- \| \| *Predictors* \| *Estimates* \| *CI* \| *p* \| \| (Intercept) \| -2.29 \| -5.49 – 0.91 \| 0.158 \| \| AQ12 \| 0.06 \| -0.01 – 0.12 \| 0.100 \| \| LackPrem \| 0.14 \| -0.01 – 0.30 \| 0.068 \| \| LackPers \| 0.11 \| -0.15 – 0.36 \| 0.401 \| \| Sbqr \| 0.02 \| -0.21 – 0.25 \| 0.872 \| \| H \| 0.24 \| 0.05 – 0.44 \| **0.013** \| \| isolation \| 0.87 \| 0.29 – 1.44 \| **0.004** \| \| R^2^ / R^2^ adjusted \| 0.337 / 0.292 \| \| \| | \|  \| **hadsAnx** \| \| \| \| --- \| --- \| --- \| --- \| \| *Predictors* \| *Estimates* \| *CI* \| *p* \| \| (Intercept) \| 1.84 \| -0.99 – 4.68 \| 0.200 \| \| NumberIsolated \| 0.58 \| 0.13 – 1.03 \| **0.011** \| \| AQ12 \| 0.08 \| 0.01 – 0.15 \| **0.019** \| \| hadsDep \| 0.20 \| -0.01 – 0.40 \| 0.062 \| \| PosUrg \| 0.13 \| -0.04 – 0.30 \| 0.142 \| \| H \| 0.19 \| 0.01 – 0.38 \| **0.041** \| \| R^2^ / R^2^ adjusted \| 0.313 / 0.275 \| \| \| |
| --- | --- | --- | --- | --- | --- | --- | --- | --- | --- | --- | --- | --- | --- | --- | --- | --- | --- | --- | --- | --- | --- | --- | --- | --- | --- | --- | --- | --- | --- | --- | --- | --- | --- | --- | --- | --- | --- | --- | --- | --- | --- | --- | --- | --- | --- | --- | --- | --- | --- | --- | --- | --- | --- | --- | --- | --- | --- | --- | --- | --- | --- | --- | --- | --- | --- | --- | --- | --- | --- | --- | --- | --- | --- | --- | --- | --- | --- | --- | --- | --- | --- | --- | --- | --- | --- | --- | --- | --- | --- | --- | --- | --- | --- | --- | --- | --- | --- | --- | --- | --- | --- | --- | --- | --- | --- | --- | --- | --- | --- | --- | --- | --- | --- | --- | --- | --- | --- | --- | --- | --- | --- | --- |

**Table E: Regression intra Gp2:**

Note: isolation: sense of loneliness, Numbercontact=number of people being in contact with, relation =impact on relational life, emotion=impact on emotional life, NegUrg=Negative Urgency, PosUrg=Positive Urgency, LackPrem=Lack of premeditation, LackPers=Lack of perseverance, H=Hopelessness, AQ12= Aggression score, NumberIsolated= Number of person being isolated with, p<.05=*, p<.005=**,p<.001=***.

| \|  \| **AQ12** \| \| \| \| --- \| --- \| --- \| --- \| \| *Predictors* \| *Estimates* \| *CI* \| *p* \| \| (Intercept) \| 23.35 \| 5.89 – 40.81 \| **0.010** \| \| hadsDep \| -0.92 \| -1.91 – 0.06 \| 0.066 \| \| age \| -0.13 \| -0.39 – 0.14 \| 0.346 \| \| hadsAnx \| 1.28 \| 0.53 – 2.04 \| **0.001** \| \| emotion \| -1.68 \| -4.35 – 0.99 \| 0.212 \| \| H \| 0.66 \| -0.01 – 1.33 \| 0.053 \| \| NegUrg \| 0.36 \| -0.13 – 0.86 \| 0.146 \| \| R^2^ / R^2^ adjusted \| 0.443 / 0.373 \| \| \| | \|  \| **hadsDep** \| \| \| \| --- \| --- \| --- \| --- \| \| *Predictors* \| *Estimates* \| *CI* \| *p* \| \| (Intercept) \| 3.44 \| -2.06 – 8.95 \| 0.214 \| \| AQ12 \| -0.07 \| -0.15 – 0.02 \| 0.121 \| \| hadsAnx \| 0.39 \| 0.15 – 0.64 \| **0.002** \| \| LackPrem \| -0.00 \| -0.21 – 0.21 \| 0.987 \| \| Sbqr \| 0.04 \| -0.18 – 0.25 \| 0.728 \| \| H \| 0.30 \| 0.11 – 0.50 \| **0.003** \| \| emotion \| -0.67 \| -1.55 – 0.21 \| 0.131 \| \| relation \| -0.39 \| -1.47 – 0.69 \| 0.467 \| \| LackPers \| 0.19 \| -0.09 – 0.48 \| 0.179 \| \| isolation \| -0.12 \| -0.90 – 0.66 \| 0.760 \| \| R^2^ / R^2^ adjusted \| 0.658 / 0.590 \| \| \| | \|  \| **hadsAnx** \| \| \| \| --- \| --- \| --- \| --- \| \| *Predictors* \| *Estimates* \| *CI* \| *p* \| \| (Intercept) \| -0.17 \| -5.98 – 5.65 \| 0.954 \| \| isolation \| 0.80 \| -0.02 – 1.62 \| 0.054 \| \| relation \| 0.02 \| -1.17 – 1.21 \| 0.975 \| \| emotion \| -0.21 \| -1.19 – 0.78 \| 0.677 \| \| AQ12 \| 0.15 \| 0.06 – 0.23 \| **0.001** \| \| Sbqr \| 0.16 \| -0.07 – 0.40 \| 0.168 \| \| hadsDep \| 0.48 \| 0.19 – 0.77 \| **0.002** \| \| H \| -0.07 \| -0.30 – 0.16 \| 0.549 \| \| R^2^ / R^2^ adjusted \| 0.637 / 0.583 \| \| \| |
| --- | --- | --- | --- | --- | --- | --- | --- | --- | --- | --- | --- | --- | --- | --- | --- | --- | --- | --- | --- | --- | --- | --- | --- | --- | --- | --- | --- | --- | --- | --- | --- | --- | --- | --- | --- | --- | --- | --- | --- | --- | --- | --- | --- | --- | --- | --- | --- | --- | --- | --- | --- | --- | --- | --- | --- | --- | --- | --- | --- | --- | --- | --- | --- | --- | --- | --- | --- | --- | --- | --- | --- | --- | --- | --- | --- | --- | --- | --- | --- | --- | --- | --- | --- | --- | --- | --- | --- | --- | --- | --- | --- | --- | --- | --- | --- | --- | --- | --- | --- | --- | --- | --- | --- | --- | --- | --- | --- | --- | --- | --- | --- | --- | --- | --- | --- | --- | --- | --- | --- | --- | --- | --- | --- | --- | --- | --- | --- | --- | --- | --- | --- | --- | --- | --- | --- | --- | --- | --- |

**Table F: Regression intra Gp 3:**

Note: isolation: sense of loneliness, Numbercontact=number of people being in contact with, relation =impact on relational life, emotion=impact on emotional life, NegUrg=Negative Urgency, PosUrg=Positive Urgency, LackPrem=Lack of premeditation, LackPers=Lack of perseverance, H=Hopelessness, AQ12= Aggression score, p<.05=*, p<.005=**,p<.001=***.
