# Supplementary material for: Psychiatric and non-psychiatric population vulnerabilities in time of a crisis: the unsuspected aggression factor
Source: BMC Psychiatry. 2023 Jun 1;23:386. doi: 10.1186/s12888-023-04843-4 (PMC10234249; doi:10.1186/s12888-023-04843-4)

For Group 1:

The analysis of the eigenvalue variance.percent cumulative.variance.percent confirmed that the very first 3 dimension may be of interest:

Dim.1 3.7097884 23.186178 23.18618

Dim.2 2.1201879 13.251174 36.43735

Dim.3 1.6528716 10.330448 46.76780

Dim.4 1.3433921 8.396201 55.16400

Dim.5 1.0871115 6.794447 61.95845

Dim.6 0.9117247 5.698280 67.65673

Dim.7 0.8302477 5.189048 72.84577

Dim.8 0.6774830 4.234269 77.08004

Dim.9 0.6099654 3.812284 80.89233

Dim.10 0.5783483 3.614677 84.50700

Dim.11 0.4653999 2.908750 87.41575

Dim.12 0.4373083 2.733177 90.14893

Dim.13 0.4265443 2.665902 92.81483

Dim.14 0.4173795 2.608622 95.42345

Dim.15 0.3774260 2.358913 97.78237

Dim.16 0.3548213 2.217633 100.00000


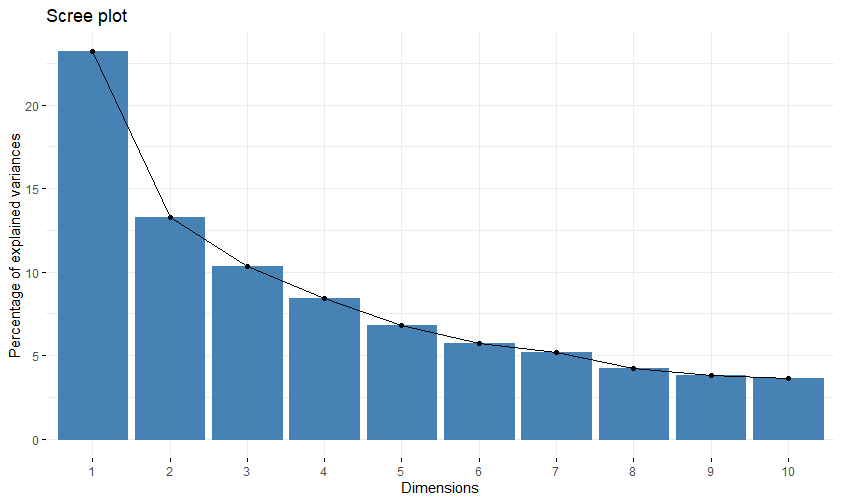


Link between the variable and the continuous variables (R-square)

=================================================================================

correlation p.value

**hadsAnx 0.7107686 3.123253e-32**

**hadsDep 0.7081095 6.674852e-32**

**AQ12 0.6878976 1.642728e-29**

H 0.6347326 4. 612782e-24

Sbqr 0.5062762 1.756694e-14

isolation 0.4830804 3.780881e-13

LackPrem 0.4738213 1.208379e-12

PosUrg 0.4733791 1.276225e-12

NegUrg 0.4635136 4.230659e-12

LackPers 0.2897307 3.021272e-05

age -0.2322222 9.090800e-04

relation -0.4358402 1.001012e-10

emotion -0.5692226 1.177776e-18

AQ12, HadsAnx, HadsDep being among the strongest concomitant variables:


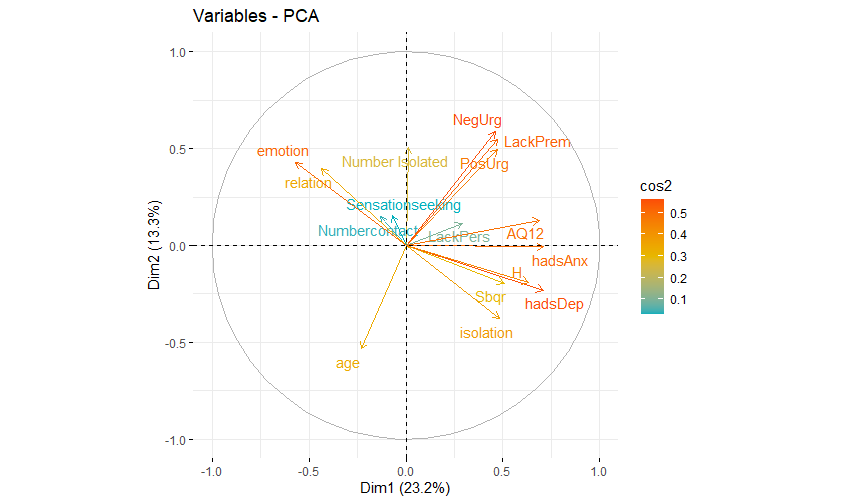


The same approaches have then been applied to Group2:

We analysed the eigenvalue variance.percent cumulative.variance.percent, leading us to consider up to 6 dimensions:

Dim.1 3.0312691 18.945432 18.94543

Dim.2 2.5772954 16.108096 35.05353

Dim.3 1.4841799 9.276124 44.32965

Dim.4 1.3000473 8.125296 52.45495

Dim.5 1.1999909 7.499943 59.95489

Dim.6 1.0272785 6.420491 66.37538

Dim.7 0.8387531 5.242207 71.61759

Dim.8 0.7499166 4.686979 76.30457

Dim.9 0.6829094 4.268184 80.57275

Dim.10 0.5973963 3.733727 84.30648

Dim.11 0.5650044 3.531277 87.83776

Dim.12 0.4772005 2.982503 90.82026

Dim.13 0.4526427 2.829017 93.64928

Dim.14 0.4292741 2.682963 96.33224

Dim.15 0.3189983 1.993740 98.32598

Dim.16 0.2678434 1.674021 100.00000


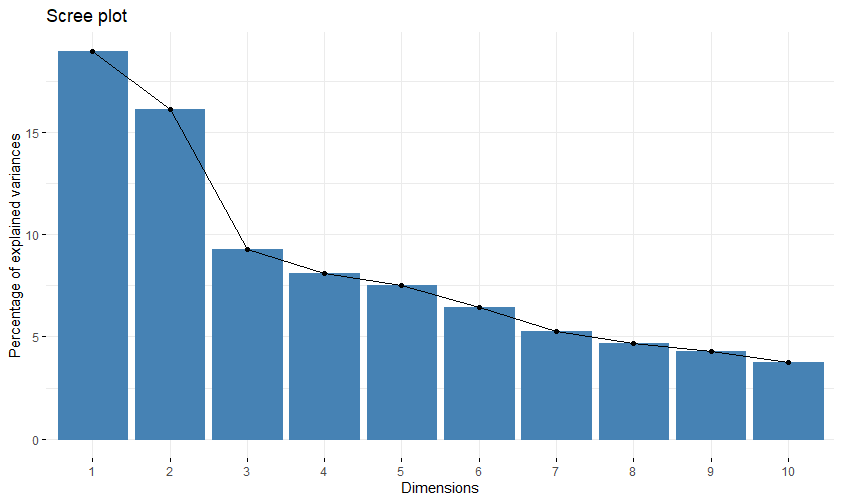


Link between the variable and the continuous variables (R-square)

=================================================================================

correlation p.value

H 0.7282435 4.272937e-20

**hadsDep 0.7038608 2.449943e-18**

isolation 0.5901237 4.866783e-12

Sbqr 0.5816378 1.152063e-11

**hadsAnx 0.5701784 3.548570e-11**

**AQ12 0.4135640 4.797261e-06**

LackPers 0.3817799 2.773999e-05

LackPrem 0.2787572 2.670876e-03

age -0.2146641 2.181738e-02

relation -0.4681722 1.499634e-07

emotion -0.5302336 1.301549e-09

With a observation here that AQ12, HadsAnxiety and HadsDep may be less important in the Gp2 than in Gp1 but still present in the dimensions of interest, but more intertwined with other variables like age, impulsivity dimensions, relation, emotions and sense of loneliness.


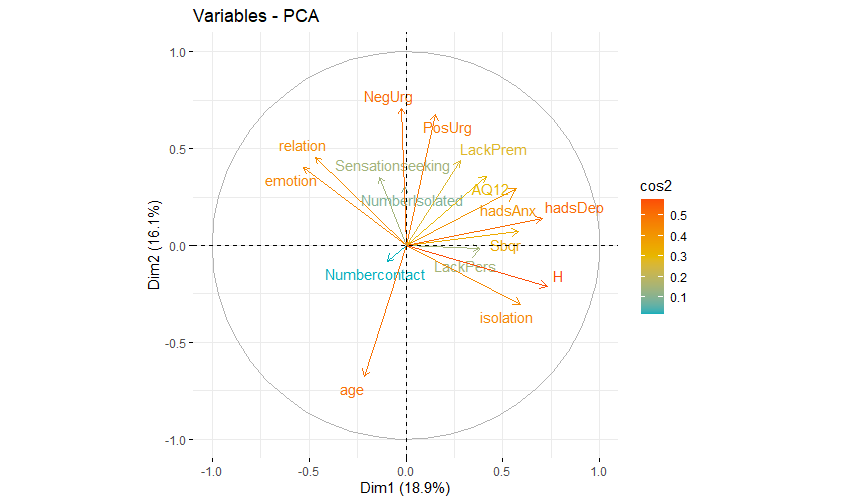


Finally, if we look into Gp3 with the PCA approach:

The analyses eigenvalue variance.percent cumulative.variance.percent lead us to consider also up to 6 Dimensions

Dim.1 4.0242161 25.151351 25.15135

Dim.2 2.3095868 14.434917 39.58627

Dim.3 1.5313801 9.571126 49.15739

Dim.4 1.2982932 8.114332 57.27173

Dim.5 1.2652349 7.907718 65.17944

Dim.6 0.9575932 5.984957 71.16440

Dim.7 0.8403810 5.252381 76.41678

Dim.8 0.7101198 4.438249 80.85503

Dim.9 0.6040840 3.775525 84.63056

Dim.10 0.5554372 3.471483 88.10204

Dim.11 0.4130707 2.581692 90.68373

Dim.12 0.3964910 2.478069 93.16180

Dim.13 0.3744655 2.340409 95.50221

Dim.14 0.3169926 1.981203 97.48341

Dim.15 0.2665605 1.666003 99.14942

Dim.16 0.1360934 0.850584 100.00000


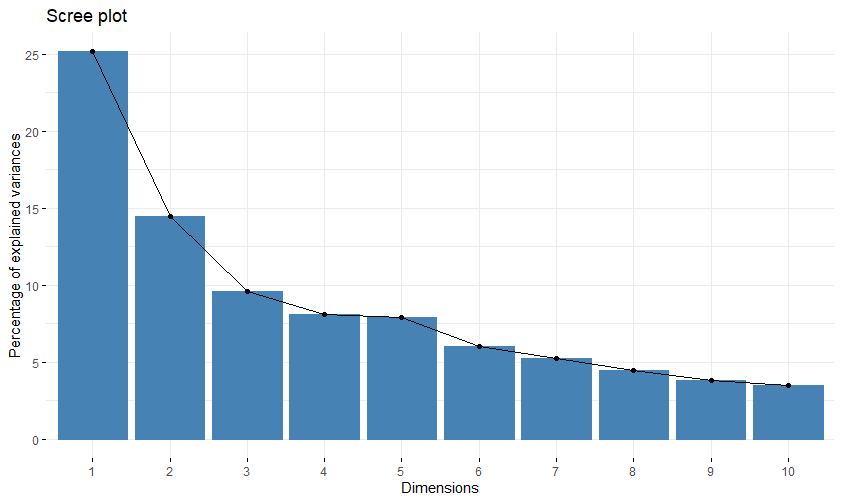


Link between the variable and the continuous variables (R-square)

=================================================================================

correlation p.value

**hadsDep 0.7934194 7.191040e-16**

**hadsAnx 0.7753685 8.354667e-15**

H 0.6625648 7.541386e-10

**AQ12 0.5657336 4.976077e-07**

Sbqr 0.5611971 6.418800e-07

LackPrem 0.5170025 6.363569e-06

isolation 0.4773461 3.856332e-05

LackPers 0.3985743 7.614155e-04

NegUrg 0.3879500 1.079874e-03

PosUrg 0.2400219 4.866683e-02

age -0.2953842 1.446603e-02

relation -0.4921733 2 .018018e-05

emotion -0.6603743 8.966876e-10

With also mitigated results on the weight of AQ12, HadsAnx and HadsDep, also among important variable but less predominantly as for Gp1


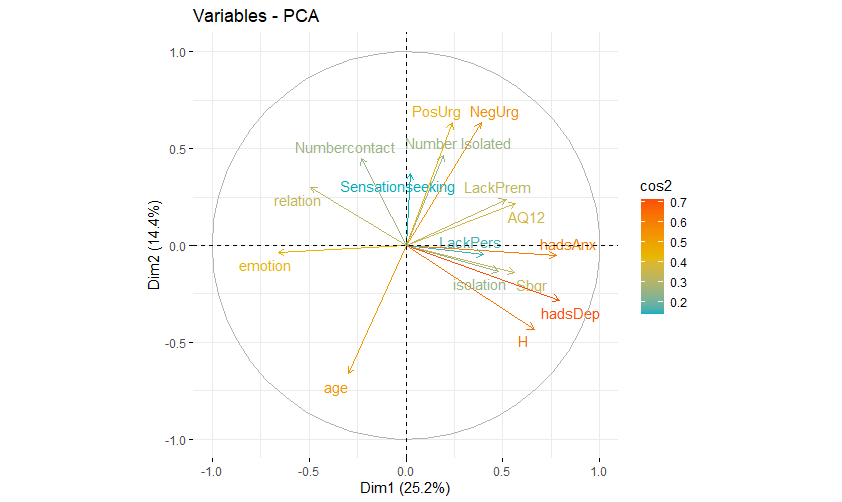

Supplement: Supplementary file 3 — Additional file 3: Complementary analysis. [file 12888_2023_4843_MOESM3_ESM.docx]
